# Stress detection using deep neural networks

**DOI:** 10.1186/s12911-020-01299-4

**Published:** 2020-12-30

**Authors:** Russell Li, Zhandong Liu

**Affiliations:** 1 St. John’s School, Houston, TX USA; 2 Department of Pediatrics, Baylor College of Medicine, Houston, TX USA; 3 Jan and Dan Duncan Neurological Research Institute, Texas Children’s Hospital, Houston, TX USA

**Keywords:** Convolutional neural network, Emotion classification, Multilayer perceptron, Stress detection

## Abstract

**Background:**

Over 70% of Americans regularly experience stress. Chronic stress results in cancer, cardiovascular disease, depression, and diabetes, and thus is deeply detrimental to physiological health and psychological wellbeing. Developing robust methods for the rapid and accurate detection of human stress is of paramount importance.

**Methods:**

Prior research has shown that analyzing physiological signals is a reliable predictor of stress. Such signals are collected from sensors that are attached to the human body. Researchers have attempted to detect stress by using traditional machine learning methods to analyze physiological signals. Results, ranging between 50 and 90% accuracy, have been mixed. A limitation of traditional machine learning algorithms is the requirement for hand-crafted features. Accuracy decreases if features are misidentified. To address this deficiency, we developed two deep neural networks: a 1-dimensional (1D) convolutional neural network and a multilayer perceptron neural network. Deep neural networks do not require hand-crafted features but instead extract features from raw data through the layers of the neural networks. The deep neural networks analyzed physiological data collected from chest-worn and wrist-worn sensors to perform two tasks. We tailored each neural network to analyze data from either the chest-worn (1D convolutional neural network) or wrist-worn (multilayer perceptron neural network) sensors. The first task was binary classification for stress detection, in which the networks differentiated between stressed and non-stressed states. The second task was 3-class classification for emotion classification, in which the networks differentiated between baseline, stressed, and amused states. The networks were trained and tested on publicly available data collected in previous studies.

**Results:**

The deep convolutional neural network achieved 99.80% and 99.55% accuracy rates for binary and 3-class classification, respectively. The deep multilayer perceptron neural network achieved 99.65% and 98.38% accuracy rates for binary and 3-class classification, respectively. The networks’ performance exhibited a significant improvement over past methods that analyzed physiological signals for both binary stress detection and 3-class emotion classification.

**Conclusions:**

We demonstrated the potential of deep neural networks for developing robust, continuous, and noninvasive methods for stress detection and emotion classification, with the end goal of improving the quality of life.

## Background

Over 70% of Americans experience stress [1]. Chronic stress results in a weakened immune system [2], cancer [3], cardiovascular disease [3, 4], depression [5], diabetes [6, 7], and substance addiction [8]. Thus, stress is deeply detrimental to physiological health and psychological wellbeing. It is of paramount importance to develop robust methods for the rapid detection of human stress. Such technologies may enable the continuous monitoring of stress. As a result, individuals may manage their daily activities to reduce stress and healthcare professionals may provide more effective treatment for stress-related illnesses. Researchers have developed different mechanisms for the detection of human stress. Tzirakis et al. [9] developed a deep neural network model that analyzed video footage to detect stress. Mirsamadi et al. [10] used a recurrent neural network that analyzed speech to detect stress.

Researchers have developed many methods to analyze physiological signals measured from sensors that are attached to the human body for stress detection and emotion classification [11]. Prior research has shown that analyzing physiological signals may reliably indicate human stress [12]. Methods based on the analysis of physiological signals offer non-invasive ways to monitor stress. As such, these methods have the potential to significantly improve humans’ quality of life. Past research on the analysis of physiological signals to detect stress has primarily used traditional machine learning approaches. The results have been mixed. In this article, we present two deep neural networks for analyzing physiological signals for stress and emotion detection that achieve an improved performance over past methods.

Much research has been conducted on using physiological signals to detect stress [13−16]. Almost all past approaches analyzed a combination of physiological signals, including signals collected from the electrocardiogram [17], electrodermal activity [18], and electromyography [19] sensors. These approaches detected stress and classified emotions by utilizing traditional machine learning algorithms to analyze physiological signals. The machine learning algorithms utilized include the decision tree, support vector machine, K-nearest neighbor, random forest, linear discriminant analysis (LDA), and others.

Healey and Picard [20] conducted one of the first studies that used physiological signals to detect the presence of human stress. The researchers used signals collected from the electrocardiogram, electromyography, electrodermal activity, and respiratory rate sensors. 22 features were hand-crafted from the aforementioned physiological signals. The LDA machine learning algorithm was used for binary classification between a stressed condition and a non-stressed condition. Gjoreski et al. [21] used a wrist-worn device that contained the accelerometer (ACC), blood volume pulse (BVP), electrodermal activity, heart rate, and skin temperature sensors. 63 features were extracted from these signals and used as inputs for the machine learning algorithm. The random forest machine learning algorithm was used for classification, and the algorithm achieved a 72% accuracy rate. Kim et al. [22] used physiological signals from the electromyography, speed and cadence, electrocardiogram, and respiratory rate sensors for emotion classification. In the experiment run by Kim et al., human participants listened to different songs in order to trigger different emotions. The researchers manually generated hand-crafted features for the machine learning algorithms and used the LDA machine learning algorithm for emotion classification. The authors achieved a subject-independent correct classification ratio of 70%. More recently, Schmidt et al. [17] conducted extensive research on stress and emotion detection using physiological signals. They investigated using physiological signals measured from sensors attached to the chest and wrist. The tasks of binary classification, which distinguished between a stressed state and a non-stressed state, and 3-class classification, which distinguished between a baseline state, a stressed state, and an amused state, were performed using multiple machine learning algorithms. For each of the two tasks, the performances of machine learning algorithms such as the decision tree, random forest, AdaBoost, LDA, and K-nearest neighbor were compared. The machine learning algorithms’ best performance for 3-class classification were 75.21% and 76.60% accuracy rates for the wrist and chest cases, respectively. The machine learning algorithms’ best performance for binary classification were 87.12% and 92.83% accuracy rates for the wrist and chest cases, respectively.

A primary drawback for all traditional machine learning approaches is the requirement for hand-crafted features to be manually generated. Almost all of previous research uses certain characteristics and statistics of the physiological signals [21, 23]. For instance, for signals collected from the electrocardiogram sensor, heart rate, heart rate variability, and related statistics including the mean, variance, and the energy of low, middle, and high frequency bands were used as features. For signals collected from the respiratory rate sensor, the mean and standard deviation of inhalation duration, exhalation duration, and respiration duration were used as features. For signals collected from the electromyography sensor, the skin conductance level and skin conductance response were extracted from the raw electromyography signals, and the related statistics for these traits such as their mean, standard deviation, and dynamic range were used as features. Computing these manually generated features is not a trivial matter since a different set of features must be manually generated from the physiological signals collected from each sensor. More importantly, these features have not been proven to accurately represent the physiological signals. There is also no guarantee that the features used by the previous approaches cover the entire feature space of the signal for machine learning algorithms being used.

## Methods

To address the challenges in manual feature engineering, we developed a deep 1D convolutional neural network and a deep multilayer perceptron neural network for stress detection and emotion classification. Instead of using hand-crafted features, the physiological signals were formatted into vectors and directly fed into the neural networks. Through supervised training, the different layers of the network learned how to represent features. The datasets from Schmidt et al. [24] were used for neural network training and testing. The deep neural networks’ performance for binary stress detection and 3-class emotion classification were compared with the best performances of the traditional machine learning algorithms used by Schmidt et al. [24]. The points of comparison between the deep neural networks and the traditional machine learning algorithms were the accuracy and F1 score of each approach.

The accuracy of a particular approach is defined as the percentage of correct predictions achieved by the approach. The equation for accuracy is shown below:

$$Accuracy = \frac{True\,Positives + True\,Negatives}{{Total\,Population}}.$$

The F1 score of a particular approach is defined as the harmonic mean of the precision and recall. The equation for the F1 score is shown below:

$$F1 \,Score = 2 \cdot \frac{Precision \cdot Recall}{{Precision + Recall}}.$$

The equations for precision and recall are shown below:

$$\begin{aligned} & Precision = \frac{True\,Positives}{{True\,Positives + False\,Positives}} \\ & Recall = \frac{True \,Positives}{{True\,Positives + False\,Negatives}}. \\ \end{aligned}$$.

### Data collection

The data analyzed in this project were downloaded from the Machine Learning Repository hosted by the University of California at Irvine. The data were made publicly available by researchers Schmidt et al. [24]. In those researchers’ experiment, 15 human participants experienced baseline, amused, and stressed conditions. The baseline condition was aimed at inducing a neutral affective state. Under the amused condition, the participants watched a series of videos designed to provoke amusement. Under the stressed condition, the participants underwent the Trier Social Stress Test [25]. During the Trier Social Stress Test, each participant was asked to perform stress-inducing tasks. These tasks included delivering a five-minute speech and counting the integers from 2023 to zero in descending steps of 17. Two datasets were collected from sensors attached to each participant’s body. The first dataset was collected from sensors attached to each participant’s chest. The sensors included the electrocardiogram sensor, electrodermal activity sensor, electromyography sensor, skin temperature sensor, respiratory rate sensor, and 3-axis accelerometer. Each sensor collected samples at a sampling rate of 700 Hz. The second dataset was collected from sensors in a wrist-worn device. The sensors included the BVP, electrodermal activity, skin temperature, and ACC sensors. The sensors collected samples at the following sampling rates: 64 Hz (BVP), 32 Hz (ACC), 4 Hz (electrodermal activity), and 4 Hz (skin temperature).

### Neural networks

Neural networks have seen growth and found success in many areas of application in recent years. Deep neural networks possess key advantages in their capabilities to model complex systems and utilize automatically learning features through multiple network layers. As such, deep neural networks are used to carry out accuracy-driven tasks such as classification and identification [26].

This article presents two deep neural networks that were developed for stress and emotion detection through the analysis of sensor-measured physiological signals. The physiological signals were directly input into the neural networks. This approach differs from traditional machine learning approaches in that traditional approaches have relied on the use of hand-crafted features as inputs.

A deep convolutional neural network primarily consists of filter layers, activation functions, pooling layers, and fully connected layers [26]. A deep convolutional neural network optimizes its parameters using supervised training. A convolution by the filtering operation and activation function is shown below:

$$\begin{aligned} & z^{\left[ l \right]} = w^{\left[ l \right]} \cdot a^{\left[ l \right]} + b^{\left[ l \right]} \\ & a^{\left[ l \right]} = g\left( {z^{\left[ l \right]} } \right) \\ \end{aligned}$$

Here ***w*** and ***a*** represent the vectors for filter coefficients and inputs, respectively. ***b*** is the bias. ***g*** represents the activation function. There are several activation functions commonly used in neural network architecture. The Rectified Linear Unit (ReLU) is one of the most popular activation functions used in neural network architecture and is defined as follows [26]:

$$g\left( z \right) = max \left( {0, z} \right).$$

The softmax function is another activation function. This function is primarily used in the last layer of a neural network that performs multi-class classification and is defined as follows:

$$g\left( z \right)_{j} = \frac{{e^{{z_{j} }} }}{{\mathop \sum \nolimits_{k = 1}^{K} e^{z} k}}.$$

In the softmax function, $z_{k}$ represents the output of the $k$th unit of the last layer in the neural network.

The sigmoid function is another activation function. It can be considered as a special case of the softmax function that is primarily used in the last layer in a neural network that performs binary classification. The sigmoid function is defined as follows:

$$g\left( z \right) = sigmoid\left( z \right) = \frac{1}{{1 + e^{ - z} }}.$$

### Deep convolutional neural network for signals from chest-worn sensors

A deep 1D convolutional neural network was developed for stress detection and emotion classification. The key reason for using a convolutional neural network was the advantage of parameter-sharing that a convolutional neural network offers. In a convolutional neural network, a small number of filters can be used for feature extraction across entire inputs. This convolutional neural network was designed to receive and analyze physiological signals from 6 sensors attached to the human chest. The sensors include the electrocardiogram, electrodermal activity, electromyography, respiratory rate, and skin temperature sensors to measure physiological information, as well as the 3-axis ACC sensor to measure 3-dimensional body movement information. The set of signals collected from each axis of the 3-axis ACC sensor was used as a separate input, so a total of 8 signals were used as inputs for the deep 1D convolutional neural network. The data collected from each of the sensors was divided into segments of window length 5 s. The data segments from all of the sensors simultaneously formed the inputs for the convolutional neural network. The convolutional neural network was designed to either detect a stressed state or a non-stressed state in a binary classification format or perform 3-class classification by distinguishing a baseline state, a stressed state, and an amused state.

The network contains 8 identical 1D convolutional blocks. Each block processes one of the 8 inputs. For 5 of the convolutional blocks, each input into 1 of the 5 blocks correlates to data collected from 1 out of the following 5 sensors: electrocardiogram, electrodermal activity, electromyography, respiratory rate, and skin temperature. For the remaining three convolutional blocks, each input into 1 of the 3 convolutional blocks correlates to physiological data collected from either the x-, y-, or z- axis of the ACC sensor.

As shown in Table 1, the 1D convolutional block consists of 3 1D convolutional layers and 3 1D max pooling layers. The first layer of the 1D convolutional block contains 8 1D filters, each with filter size of 15 and stride size of 2. Each filter output uses the ReLU activation function. The layer is followed by a 1D max pooling layer with window size of 4 and stride size of 4. The second layer of the 1D convolutional block contains 16 1D filters, each with filter size of 7 and stride size of 2. Each filter output uses the ReLU activation function. The layer is followed by a max pooling layer with window size of 4 and stride size of 4. The third layer of the 1D convolutional block contains 32 1D filters, each with filter size of 3 and stride size of 1. Each filter output uses the ReLU activation function. The layer is followed by a max pooling layer with window size of 2 and stride size of 2. Data is input into the convolutional block in the form of a 3500 × 1 vector. The outputs of the convolutional block are 32 17 × 1 vectors.

**Table 1:** Configuration of 1D convolutional block

	Layer 1	Layer 2	Layer 3
Number of filters	8	16	32
Filter size	32	7	3
Filter stride	2	2	1
Activation function	ReLU	ReLU	ReLU
Pooling size	4	4	2
Pooling Stride	4	4	2

**Fig. 1:**
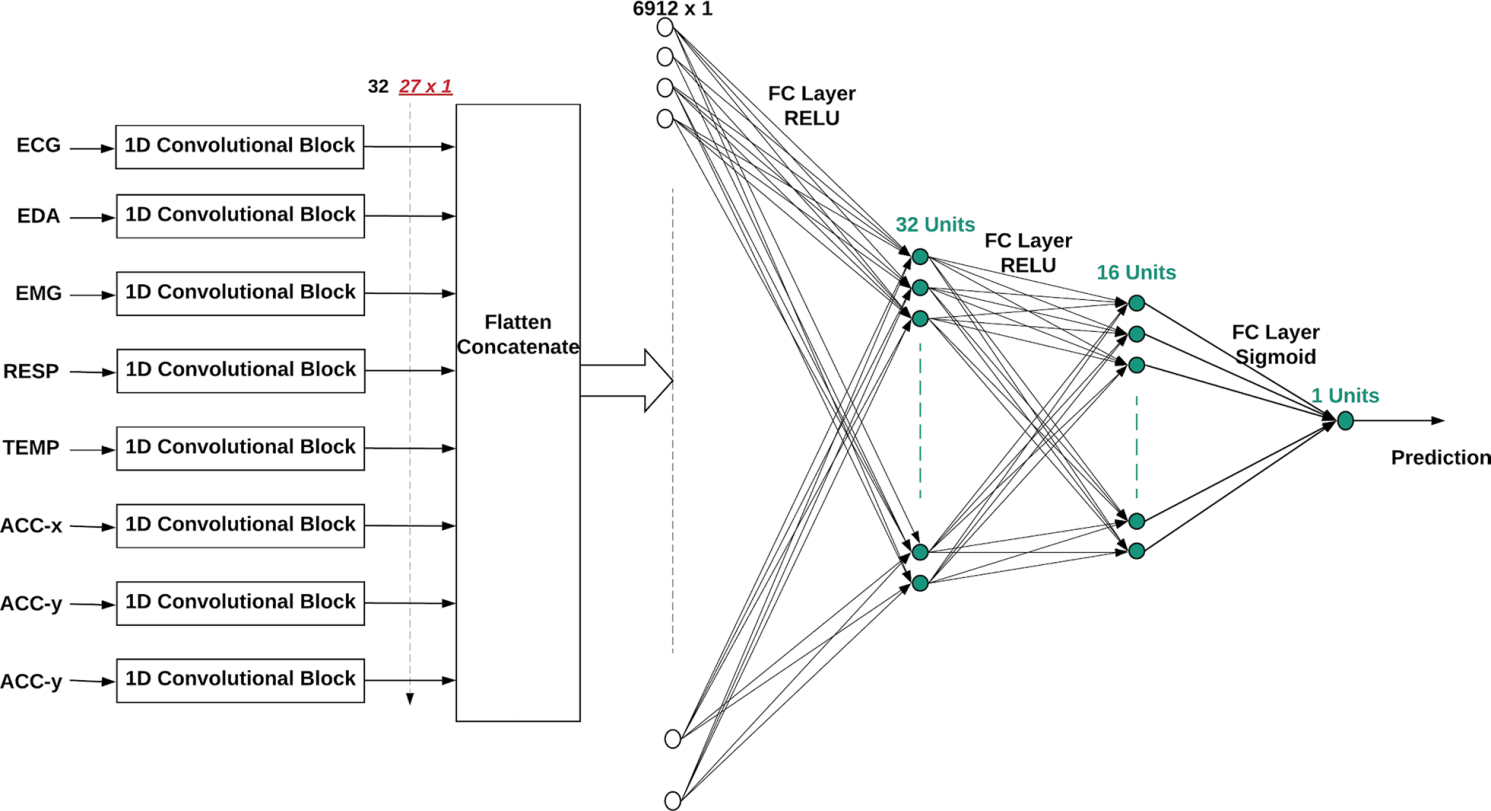
The diagram of the proposed deep 1D convolutional neural network

**Fig. 2:**
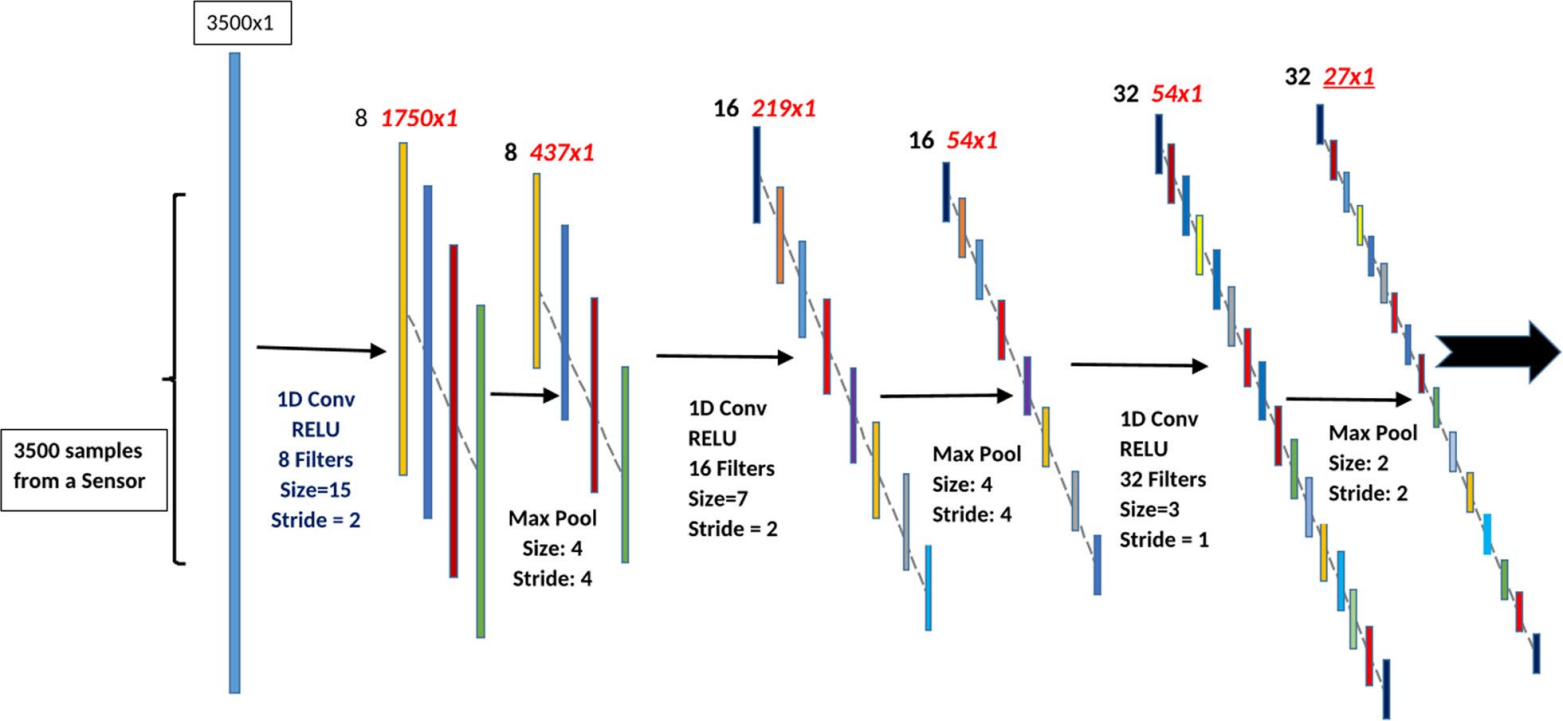
The diagram of one block in the proposed deep 1D convolutional neural network

As shown in Fig. 1, the deep convolutional neural network contains 3 fully connected layers after the 1D convolutional blocks. The data from all the sensors are combined by flattening all the output vectors from each 1D convolutional block (Fig. 2) and all the data are concatenated into one vector, which is fed into a fully connected layer with 32 units. Each unit uses the ReLU activation function. The first layer is followed by the second fully connected layer with 16 hidden units. Each unit uses the ReLU activation function. The last fully connected layer is the output layer. In the case of binary stress detection, it has one hidden unit, using the sigmoid as its activation function. For three class emotion detection, the last layer has three hidden units, all using the softmax activation function.

### Multilayer perceptron neural network for signals from wrist-worn sensors

**Fig. 3:**
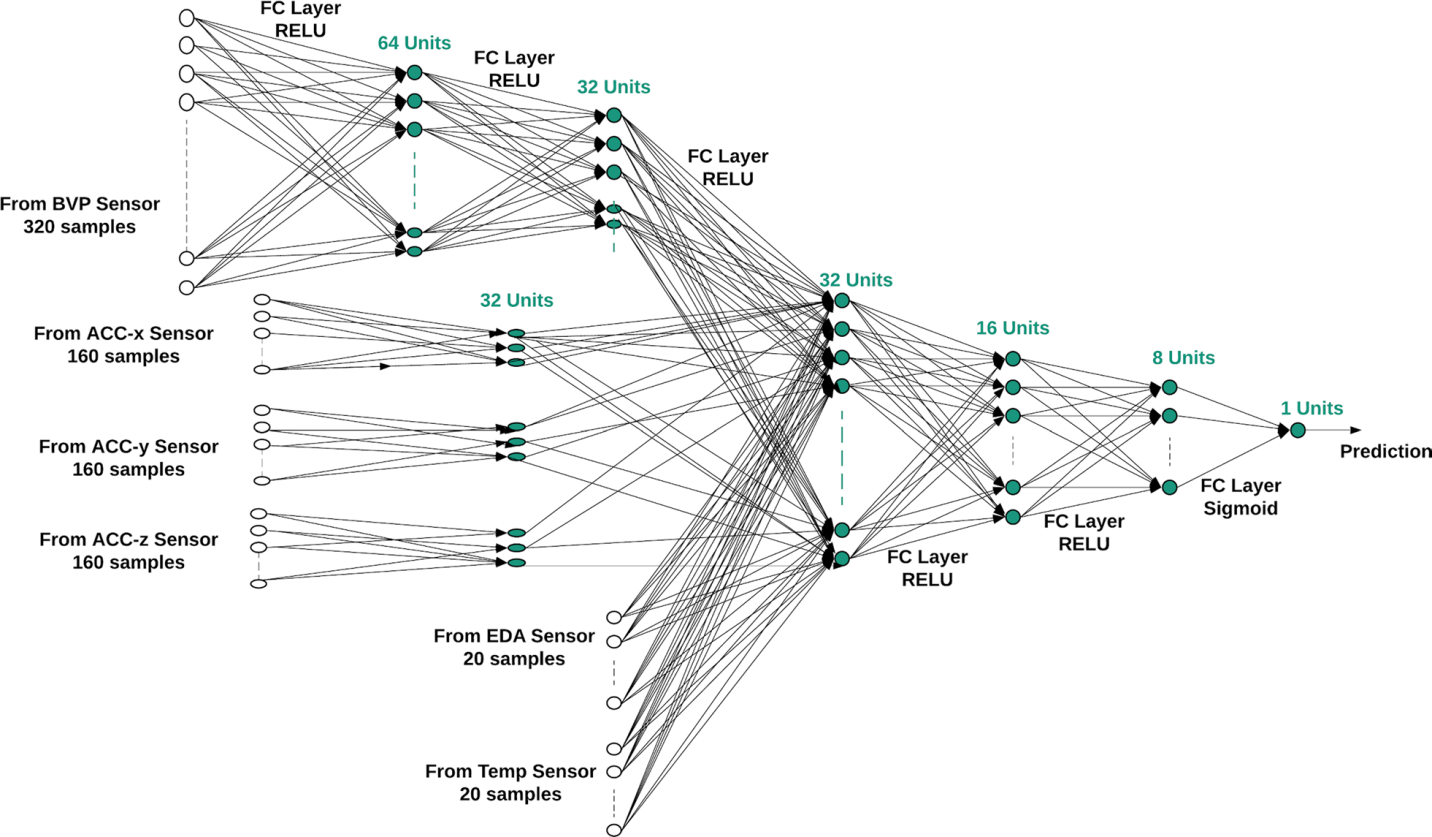
The diagram of the proposed multilayer perceptron neural network

The sensors on the wrist-worn device include BVP, electrodermal activity, skin temperature, and ACC. One key difference between the chest-worn and wrist-worn sensors is the size of the input signals from the sensors. The inputs from wrist-worn sensors are sampled at a much lower sampling rate, which results in a much smaller input size. The small input size makes using a multilayer perceptron network realistic, since the network will not have to perform as many calculations as a network faced with a larger input size. Another difference between the chest-worn and wrist-worn sensors is that the signals measured from the sensors attached to the wrist have different sampling frequencies. Thus, the neural network analyzing the data collected from wrist-worn sensors should be designed to handle multiple sensor inputs, each with a different number of inputs. We designed a multilayer perceptron neural network for processing physiological signals from wrist-worn sensors (Fig. 3).

As shown in Fig. 3, the data from sensors BVP and ACC are fed into their respective fully connected layers. The data from the BVP sensor goes through 2 hidden layers with 64 and 32 hidden units, while the data from the ACC sensor goes through 1 hidden layer with 32 hidden units. Each hidden unit uses the ReLU activation function. The outputs of the BVP and ACC sensors from their respective hidden layers are concatenated with signals from the electrodermal activity and temperature sensors and fed into three consecutive fully connected layers. For the first two of these hidden layers, each unit uses the ReLU activation function. Depending on the task being performed by the network (emotion classification vs. stress detection), the third hidden layer will use a different activation function. In the case of 3-class emotional classification, the layer, which maps from 8 hidden units to 3 hidden units, uses the softmax activation function. In the case of stress detection, the layer, which maps from 8 hidden units to 1 hidden unit, uses the sigmoid activation function.

### Neural network training

The major components related to the training of our neural networks are as follows:

- Training and Testing Data: The entire dataset was randomly scrambled and divided into a training dataset and a testing dataset with a 7 to 3 ratio.
- Optimization: Adam
- Loss Function (Binary Stress Detection):
  - Binary Cross Entropy
- Loss Function (3-Class Emotion Classification):
  - Categorical Cross Entropy
- Epoch Size: 100
- Batch Size: 40
- Validation: tenfold cross validation
- Deep Learning Library: Keras, run on Google Colab

## Results

The performances of the two trained deep neural networks with regard to binary stress detection and 3-class emotion classification were evaluated. The performances were evaluated for two cases: a case in which the signals from the 3-axis ACC sensor were included and a case in which the signals from the 3-axis ACC sensor were not included. The test dataset was used for all of the performance evaluations. The results are compared with the best results of machine learning methods evaluated in the work of Schmidt et al. [11]. The key reason for comparing our study to the work of Schmidt et al. is that both the work of those resources and our work are based on the same data set. As such, this allows for apples-to-apples comparison. In addition, Schmidt et al.’s work is comprehensive in terms of the machine learning algorithms examined, and their results are comparable to what has been reported recently on using machine learning approaches for stress detection [16].

In both cases evaluated for 3-class emotion classification, using signals from chest-worn sensors, the proposed deep 1D convolutional neural network outperformed the best performance of the traditional machine learning algorithms as reported by Schmidt et al. For the case of using all physiological signals, including signals from the ACC sensor, the proposed deep neural network achieved an accuracy rate of 99.55% and an F1 score of 99.46%. This compares to the accuracy rate of 76.50% and the F1 score of 72.49% achieved by the LDA machine learning algorithm as reported by Schmidt et al. For the case of using all physiological signals except signals from the ACC sensor, the proposed deep neural network achieved an accuracy rate of 97.48% and an F1 score of 96.82%. This compares to the accuracy rate of 80.34% and the F1 score of 72.51% achieved by the AdaBoost machine learning algorithm as reported by Schmidt et al.

In both cases evaluated for 3-class emotion classification, using signals from wrist-worn sensors, the proposed deep multilayer perceptron neural network outperformed the best performance of the traditional machine learning algorithms as reported by Schmidt et al. For the case of using all physiological signals, including signals from the ACC sensor, the proposed deep neural network achieved an accuracy rate of 98.38% and an F1 score of 97.96%. This compares to the accuracy rate of 75.21% and the F1 score of 64.12% achieved by the AdaBoost machine learning algorithm as reported by Schmidt et al. For the case of using all physiological signals except signals from the ACC sensor, the proposed deep neural network achieved an accuracy rate of 93.64% and an F1 score of 92.44%. This compares to the accuracy rate of 76.17% and the F1 score of 66.33% achieved by the Random Forest machine learning algorithm as reported by Schmidt et al.

In both cases evaluated for binary stress detection, using signals from chest-worn sensors, the proposed deep 1D convolutional neural network outperformed the best performance of the traditional machine learning algorithms as reported by Schmidt et al. For the case of using all physiological signals, including signals from the ACC sensor, the proposed deep neural network achieved an accuracy rate of 99.80% and an F1 score of 99.67%. This compares to the accuracy rate of 92.83% and the F1 score of 91.07% achieved by the LDA machine learning algorithm as reported by Schmidt et al. For the case of using all physiological signals except signals from the ACC sensor, the proposed deep neural network achieved an accuracy rate of 99.14% and an F1 score of 98.61%. This compares to the accuracy rate of 93.12% and the F1 score of 91.47% achieved by the LDA machine learning algorithm as reported by Schmidt et al.

In both cases evaluated for binary stress detection, using signals from wrist-worn sensors, the proposed deep multilayer perceptron neural network outperformed the best performance of the traditional machine learning algorithms as reported by Schmidt et al. For the case of using all physiological signals, including signals from the ACC sensor, the proposed deep neural network achieved an accuracy rate of 99.65% and an F1 score of 99.42%. This compares to the accuracy rate of 87.12% and the F1 score of 84.11% achieved by the Random Forest machine learning algorithm as reported by Schmidt et al. For the case of using all physiological signals except signals from the ACC sensor, the proposed deep neural network achieved an accuracy rate of 97.62% and an F1 score of 96.18%. This compares to the accuracy rate of 88.33% and the F1 score of 86.10% achieved by the LDA machine learning algorithm as reported by Schmidt et al.

## Discussion

### Comparison between deep neural networks and traditional machine learning algorithms

The results shown in Tables 2, 3, 4 and 5 demonstrate that the deep 1D convolutional neural network and deep multilayer perceptron neural network achieve superior performance over traditional machine learning approaches. The networks have higher accuracy rates and F1 scores for both binary stress detection and 3-class emotion classification. The superior performance was achieved in both the case of using all physiological signals, including signals from the 3-axis ACC sensor, and the case of using all physiological signals except signals from the 3-axis ACC sensor. The deep neural networks’ superiority to traditional machine learning algorithms was demonstrated in multiple aspects.

**Table 2:** Performance comparison for emotion classification from chest-measured signals

	Best performance of Schmidt et al		Performance of deep 1D convolutional neural network	
ML algorithm	LDA	AdaBoost		
	Including ACC sensor (%)	Not including ACC sensor (%)	Including ACC sensor (%)	Not including ACC sensor (%)
Accuracy	76.50	80.34	99.55	97.48
F1 Score	72.49	72.51	99.46	96.82

**Table 3:** Performance comparison for emotion classification from wrist-measured signals

	Best performance of Schmidt et al		Performance of multilayer perceptron neural network	
ML algorithm	AdaBoost	Random forest		
	Including ACC sensor (%)	Not including ACC sensor (%)	Including ACC sensor (%)	Not including ACC sensor (%)
Accuracy	75.21	76.17	98.38	93.64
F1 Score	64.12	66.33	97.96	92.44

**Table 4:** Performance comparison for stress detection from chest-measured signals

	Best performance of Schmidt et al		Performance of deep 1D convolutional neural network	
ML algorithm	LDA	LDA		
	Including ACC sensor (%)	Not including ACC sensor (%)	Including ACC sensor (%)	Not including ACC sensor (%)
Accuracy	92.83	93.12	99.80	99.14
F1 Score	91.07	91.47	99.67	98.61

**Table 5:** Performance comparison for stress detection from wrist-measured signals

	Best performance of Schmidt et al		Performance of multilayer perceptron neural network	
ML algorithm	Random forest	Random forest		
	Including ACC sensor (%)	Not including ACC sensor (%)	Including ACC sensor (%)	Not including ACC sensor (%)
Accuracy	87.12	88.33	99.65	97.62
F1 score	84.11	86.10	99.42	96.18

First, the two deep neural networks performed significantly better on both tasks than traditional machine learning algorithms. Both of the deep neural networks achieved between 6 and 12% improvement over traditional machine learning algorithms in terms of accuracy for binary stress detection. The accuracy improvement of the deep neural networks over traditional machine learning algorithms in 3-class emotion classification was even more prominent. Both of the deep neural networks achieved between 17 and 23% improvement in terms of accuracy for 3-class emotion classification. For both tasks, the superior performance represents both the case of using physiological signals without the 3-axis ACC sensor and the case of using physiological signals with the 3-axis ACC sensor.

Second, the two deep neural networks are far more architecturally consistent than traditional machine learning algorithms. For either the chest or wrist cases, only one neural network is able to carry binary stress detection and 3-class emotion classification successfully with consistent superior performance. The only difference in the neural networks between binary stress detection and 3-class emotion classification is the composition of the last layer in each deep neural network. In binary stress detection, the sigmoid activation function is used in the last layer, so the last layer of each deep neural network has 1 unit. In three-class emotion classification, the softmax activation function is used in the last layer, so the last layer of each deep neural network has 3 units. This architectural consistency contrasts with that of the traditional machine learning algorithms, as reported by Schmidt et al. [11] and shown in Tables 2, 3, 4 and 5. The tables indicate that different traditional machine learning algorithms achieved best performance under different configurations. For instance, for the task of 3-class emotion classification, based on the analysis of physiological signals from a wrist-worn device, the random forest algorithm achieved the best performance for analyzing physiological signals, not including signals collected from the 3-axis ACC sensor, whereas the AdaBoost algorithm achieved the best performance for analyzing physiological signals, including signals collected from the 3-axis ACC sensor. The downside of this trait of the traditional machine learning algorithms is that developing a real-world product for stress detection and emotion classification would be challenging because multiple machine learning models would need to be implemented to handle different types of tasks. Therefore, the two deep neural networks may be more suited towards real-world application than traditional machine learning algorithms.

Third, the two deep neural networks consistently demonstrate high performance. A slight performance drop between binary stress detection and 3-class emotion classification is expected, as multi-class classification is a more challenging task than binary classification. As shown in Tables 2, 3, 4 and 5, the performance drop, in terms of accuracy, from binary stress detection to 3-class emotion classification, ranges from 0.25 to 3.98%. On the other hand, traditional machine learning algorithms do not consistently demonstrate high performance. The accuracy rates of the traditional algorithms drop more than 10% when switching from binary stress detection to 3-class emotion classification. For both the deep 1D convolutional neural network and the deep multilayer perceptron neural network, virtually an identical network structure is used for both binary stress detection and 3-class emotion classification, except for the number of activation units and the activation function used in the last layer of each neural network. Thus, the consistent performance demonstrated by the two deep neural networks for both binary stress detection and 3-class emotion classification indicates that the two neural networks are able to “learn” the underlying features of the physiological signals relatively well.

Fourth, the neural networks’ performance improves when the signals from the 3-axis ACC sensor are added to the inputs of each neural network, as shown in Tables 2, 3, 4 and 5. This is in contrast to the performance drop for the machine learning algorithms, as shown in Tables 2, 3, 4 and 5. The performance drop of the traditional machine learning algorithms occurs for both binary stress detection and 3-class emotion classification, measuring physiological data from both the chest-worn sensors and the wrist-worn sensors. This performance drop ranges from 0.29% to 3.84%. However, adding signals from the ACC sensor should improve the performance of an ideal model, as more data points are being analyzed in the tasks performed. Thus, the two deep neural networks, which appropriately utilize the information gathered by the additional ACC sensor, are likely superior models to the traditional machine learning algorithms.

### Comparison of the two deep neural networks

Tables 2, 3, 4 and 5 also provide a comparison of the two deep neural networks developed in this experiment. The tables indicate that the deep 1D convolutional neural network, which analyzed physiological signals from chest-worn sensors, performed marginally better than the deep multilayer perceptron neural network, which analyzed physiological signals from wrist-worn sensors. This was expected for two reasons. First, the total number of physiological signals being input into the deep 1D convolutional neural network was higher than the total number of physiological signals being input into the multilayer perceptron neural network. Second, the signals from the chest-worn sensors are sampled at much higher frequencies and so are of a higher quality. Thus, the deep 1D convolutional network processed a greater amount of higher quality data, implying that the network would perform better than the deep multilayer perceptron neural network. Nevertheless, even with a fewer number of less frequently sampled signals being input, the multilayer perceptron neural network’s high performance demonstrates the network’s capabilities.

### Limitations and implications for future research

To extend on this experiment in future research, the two neural networks must be trained and tested on much larger datasets with diverse human populations. This would increase the robustness of the networks, as they would be exposed to a more accurate representation of the overall human population. The datasets used in this project were collected from 15 human participants [11], which may not adequately represent the overall human population. The rationale for exposing the neural networks to a dataset representative of the entire human population is that the sensitivity level of stress conditions (i.e., under what circumstances a person experiences stress) is individual-based and varies from person to person.

## Conclusions

We developed two deep neural networks: a deep 1D convolutional neural network and a deep multilayer perceptron neural network. The networks analyzed physiological signals measured from chest-worn and wrist-worn sensors to perform the two tasks of binary stress detection and 3-class emotion classification. The performance of the two deep neural networks were evaluated and compared with that of traditional machine learning algorithms used in previous research [11]. The results indicate that the two deep neural networks performed significantly better for both tasks than the traditional machine learning algorithms. We demonstrated the potential of deep neural networks for developing robust, continuous, and noninvasive methods for stress detection and emotion classification, with the end goal of improving the quality of life.

## Data Availability

The datasets which were analyzed in the current study (WESAD) are publicly accessible in the University of California at Irvine Machine Learning Repository, https://archive.ics.uci.edu/ml/datasets/WESAD+%28Wearable+Stress+and+Affect+Detection%29.

## Abbreviations

1D: 1-Dimensional
LDA: Linear discriminant analysis
ACC: Accelerometer
BVP: Blood volume pulse
ReLU: Rectified Linear Unit
